# A novel dataset of date fruit for inspection and classification

**DOI:** 10.1016/j.dib.2023.110026

**Published:** 2024-01-02

**Authors:** Abdul Khalique Maitlo, Riaz Ahmed Shaikh, Rafaqat Hussain Arain

**Affiliations:** Institute of Computer Science, Shah Abdul Latif University Khairpur, Pakistan

**Keywords:** Artificial Intelligence, Machine learning, Deep learning, Classification, Agriculture science

## Abstract

Date fruit grading and inspection is a challenging and crucial process in the industry. The grading process requires skilled and experienced labour. Moreover, the labour turnover in the date processing industries has been increased regularly. Therefore, due to the lack of trained labour, the quality of date fruit is often compromised. It leads to fruit wastage and instability of fruit prices. Currently, deep learning algorithms have achieved the research community's attention in solving the problems in the agriculture sector. The pre-trained models like VGG16 and VGG19 have been applied for the classification of date fruit [1,2].

Furthermore, machine learning techniques like K-Nearest Neighbors, Support Vector Machine, Random Forest and a few others [3], [4], [5], [6] have been used for grading of date fruit. Therefore, classification and sorting of date fruit problems have become common in the industry. The classification and grading of date fruit needed a neat and clean dataset. In this article, an indigenous and state-of-the-art dataset of date fruit is offered. The dataset contains images of four date fruit varieties. It consists of 3004 pre-processed images of different classes and grades.

Moreover, images have been sorted based on size as large, medium, and small. Additionally, it is graded based on the quality as grade 1, grade 2, and grade 3. This dataset is separated into eighteen different directories for the facilitation of the researchers. It may contribute to develop an intelligent system to grade and inspect date fruit. This system may add value to the sustainable economic growth of fruit processing industries and farmers locally and internationally.

Specifications Table

**Table:** 

Subject	Computer Science, Agriculture Science, Machine learning
Specific subject area	Computer vision, Image processing, Image classification
Data format	Raw, Analyzed
Type of data	Images
Data collection	The dataset has been collected in a controlled environment through a ring light with a 26 cm outer diameter and a colour temperature of 3200K-5600K. The Huawei Y6s model JAT-L29 with 13 13-megapixel camera has been used to acquire images of different varieties of date fruit. Samples of date fruit have been collected from the fields and local industry of Khairpur Mirs’. Date fruits were examined with the help of industry experts and local farmers
Data source location	Date fruit collected from village Pir Bux Maitlo and local industry of the Khairpur Mirs' o Sindh Pakistan
Data accessibility	Repository name: Mandalay Data Data identification number: 10.17632/s5zfvsw5kv.3 Direct URL to data: https://data.mendeley.com/datasets/s5zfvsw5kv/3

## 1 Value of the Data

• Date fruit processing is one of the most challenging, time-consuming, and intensive tasks. A machine learning-based model is used to sort and classify the date fruit.
• The fruit processing intelligent system can help reduce food waste, increase sustainable growth, and enhance quality production that stable farmers economically.
• The dataset is beneficial for the researchers to conduct the studies to develop a novel system for date fruit processing. Additionally, researchers will perform experiments and testing along with other date fruit varieties. It can enhance the classification of different varieties of date fruits, like the work published by other researchers [7,8].

## 2 Data Description

This dataset contains images of four date fruit varieties. The dataset images have been organized according to the developed standards of the international market [9]. The Date fruit folder comprises four sub-folders of varieties (Aseel, Fasli toto, Gajar and Kupro). Aseel and Fasli toto date fruit folders contain large, medium, and small sub-folders. Furthermore, these sub-folders contain a directory of grade 1, in which images of A quality grade are stored. The Gajar date fruit variety folder contains three sub-folders depending on size standard (large, medium, and small). The quality-based classes like Grade-1, Grade-2, and Grade-3 sub-folders are stored in these folders.

**Fig. 1:**
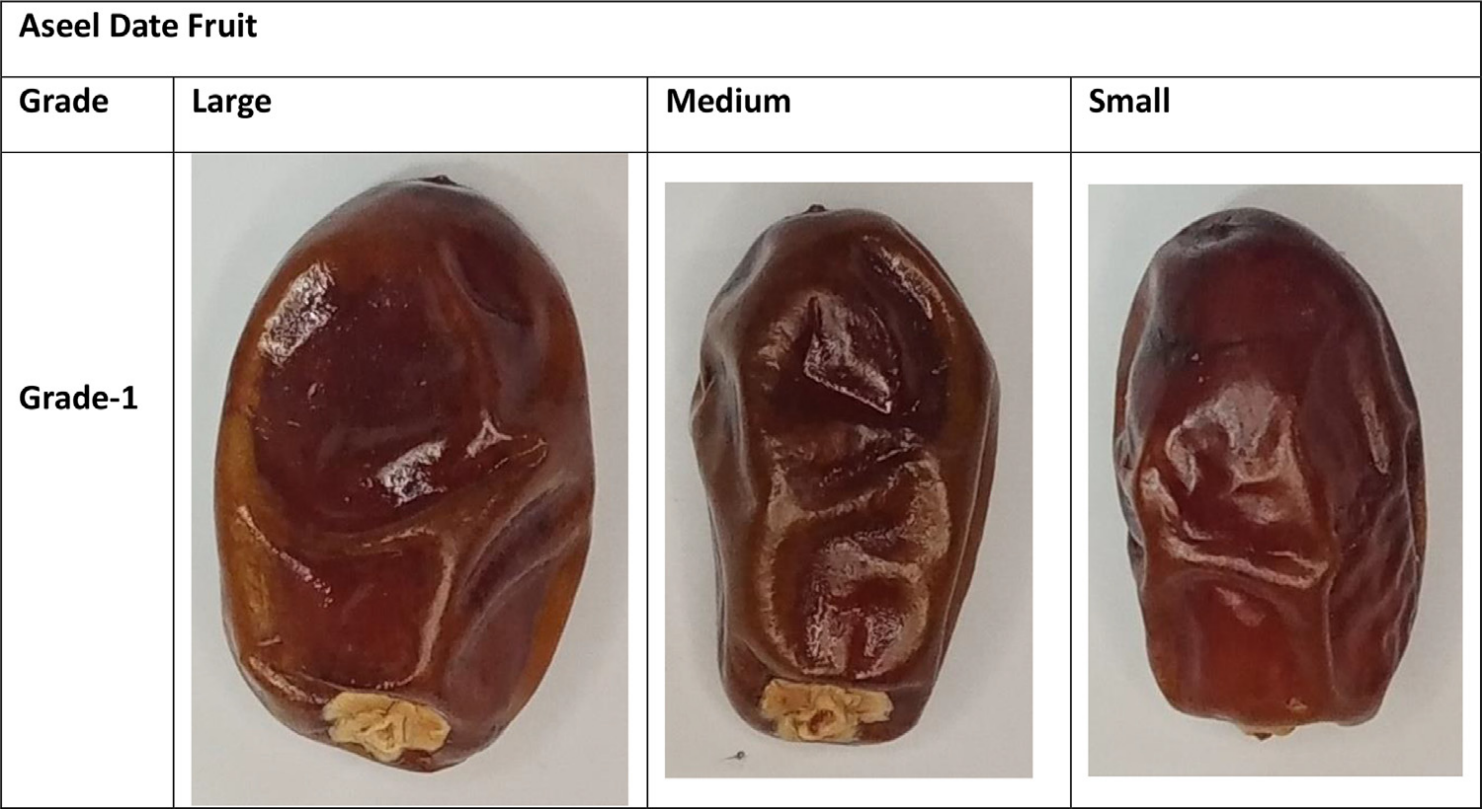
Sample of Aseel Date Fruit.

**Fig. 2:**
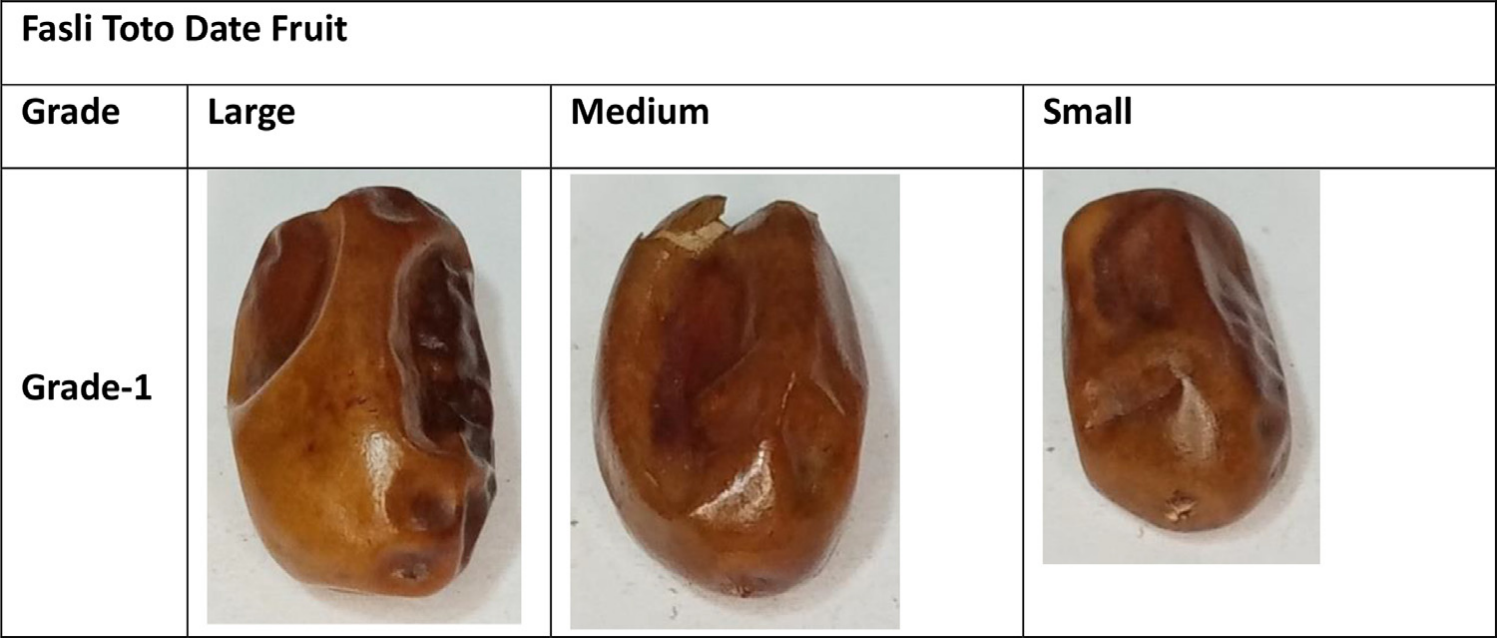
Sample of Fasli Toto.

**Fig. 3:**
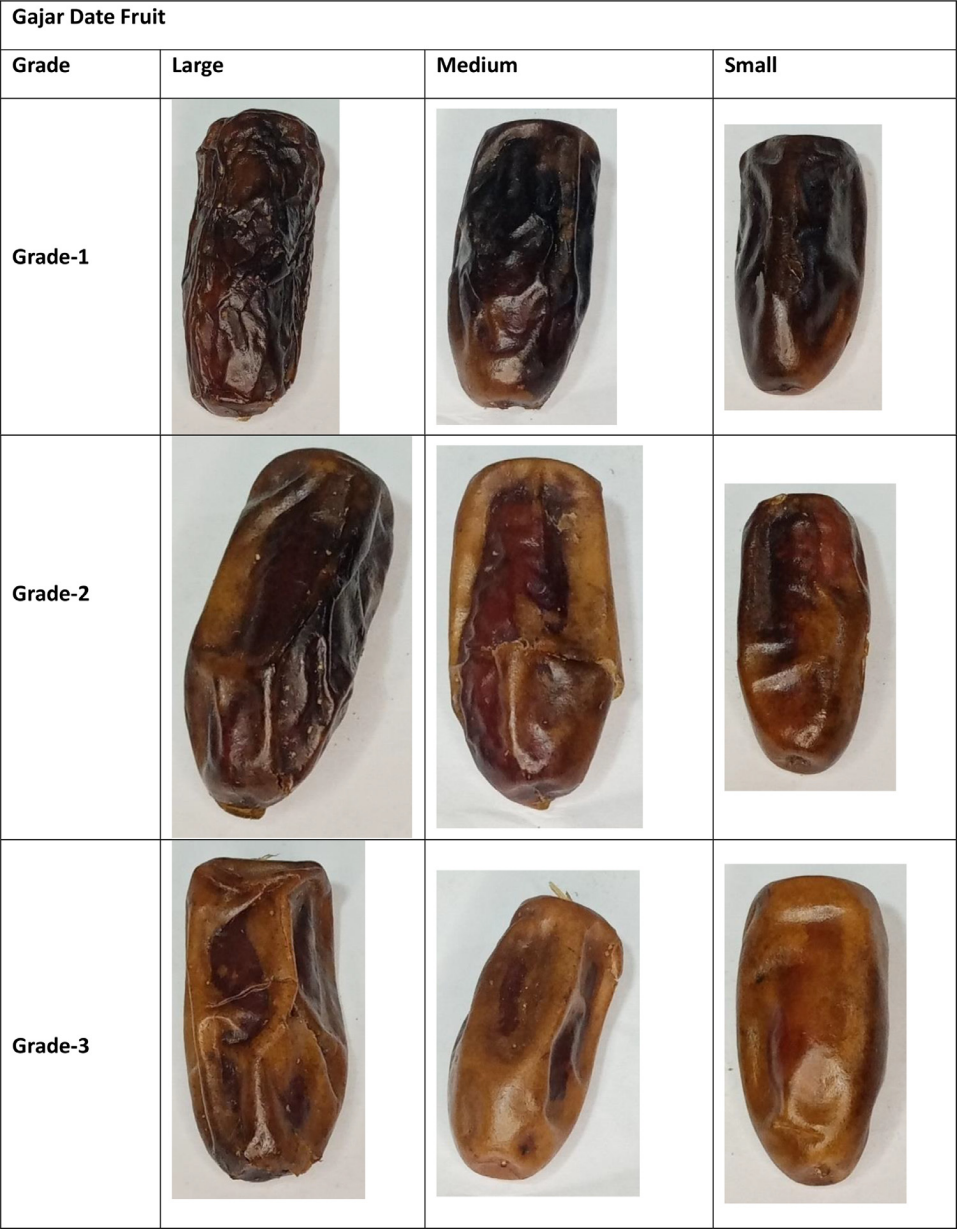
Sample of Gajar Date Fruit.

**Fig. 4:**
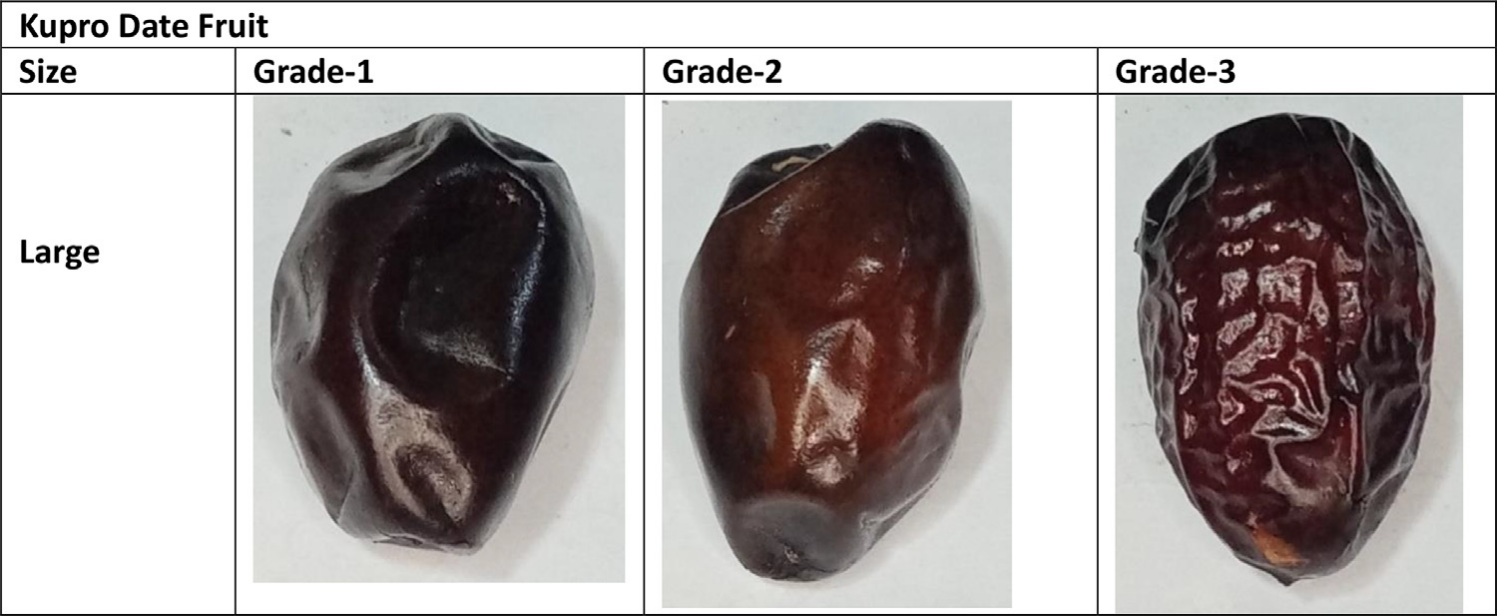
Sample of Kupro Date Fruit.

Additionally, the images of the large category of Kupro date fruit variety have been stored in a large directory. The large directory consists of three quality-based sub-folders. The quality-based grade names are mentioned above. The samples of the collected dataset are mentioned in Fig. 1, Fig. 2, Fig. 3, Fig. 4.

## 3 Experimental Design, Materials and Methods

### 3.1 Dataset sample collection

The date fruit variety samples were collected from the field and local industry of Khairpur Mirs'. The lengths of samples of date fruit varieties were different. A random sample of Aseel 500, Fasli Toto 550, Gajar 700, and Kupro 500 were collected. The collected samples were examined with the help of local industry experts and farmers. The experts and farmers separated date fruits by size, colour, texture, and shape.

### 3.2 Setup of controlled environment

The local industry experts and farmers have completed the date fruit varieties shortlisting process. The shortlisted date fruits were ready for the image acquisition process. The controlled environment-based setup was established for image acquisition. The environment comprises the Huawei Y6s model JAT-L29 with a 13-megapixel camera and ring light. The ring light configuration was 26 cm outer diameter, a ring light having 3200K-5600K colour temperature with white light. Specifically, images were acquired through the top view with a 25.4 cm distance between the camera and the object. The acquired images were stored in their respective folders. Moreover, the captured images were verified manually to remove blurred and poor-quality images. The setup process has been utilized by the author [10].

### 3.3 Preprocessing of dataset

The verified dataset has been preprocessed. The preprocessing operations are image enhancement, edge detection, and object extraction. These operations have been performed using Python language script and the OpenCV library. The image enhancement was performed using a Gaussian filter. Furthermore, edges were detected with the help of the Canny Edge detector function.

Moreover, a morphological kernel was created using morphological structuring with 7,7 values. The detected edges and morphological kernel passed for morphological transformations using the opening operation to extract object shapes [11]. The object was also successfully extracted from the raw image and formed a new image. The formed image of the object has been stored in respective directories.

Overall, 3004 images have been stored in the dataset. Each image of the dataset has a different size. The distribution dataset images are mentioned in Table 1, Table 2, Table 3.

**Table 1:** Grade-1 date fruit detail.

Grade-1				
Date fruit varieties	Large	Medium	Small	Sub total
**Aseel**	155	168	171	494
**Fasli Toto**	153	153	84	390
**Gajar**	175	201	200	576
**Kupro**	324	0	0	324
			**Total**	1784

**Table 2:** Grade-2 date fruit detail.

Grade-2				
Date fruit varieties	Large	Medium	Small	Sub total
**Gajar**	120	62	141	323
**Kupro**	370	0	0	370
			**Total**	693

**Table 3:** Grade-3 date fruit detail.

Grade-3				
Date fruit varieties	Large	Medium	Small	Sub total
**Gajar**	122	80	209	411
**Kupro**	116	0	0	116
			**Total**	527

### 3.4 Augmented dataset

The augmentation process has been completed to increase the length of the dataset. Moreover, the dataset sample collection exercise has remained complex and challenging due to less quantity was available in the industry. Therefore, augmentation techniques have been performed to overcome the smaller number of dataset images. The rotation function through different values has been utilized to develop an impactful dataset with 19229 images for the training and testing of deep learning models. The augmented dataset details are given in Table 4, Table 5, Table 6.

**Table 4:** Grade-1 date fruit detail of augmented dataset.

Grade-1				
Date fruit varieties	Large	Medium	Small	Sub total
**Aseel**	1240	1344	1368	3952
**Fasli Toto**	1224	1224	924	3372
**Gajar**	1200	1200	1000	3400
**Kupro**	1000	0	0	1000
			**Total**	11,724

**Table 5:** Grade-2 date fruit detail of augmented dataset.

Grade-2				
Date Fruit Varieties	Large	Medium	Small	Sub Total
**Gajar**	960	789	1128	2877
**Kupro**	1100	0	0	1100
			**Total**	3977

**Table 6:** Grade-3 date fruit detail of augmented dataset.

Grade-3				
Date Fruit Varieties	Large	Medium	Small	Sub Total
**Gajar**	976	640	1100	2716
**Kupro**	812	0	0	812
			**Total**	3528

## Limitations

Not applicable.

## Ethics Statement

The funding was not received from any agency or organization. Therefore, no conflict of interest. Also, both humans and animals were not involved in the experiment.

## CRediT authorship contribution statement

**Abdul Khalique Maitlo:** Methodology, Data curation, Investigation, Validation. **Riaz Ahmed Shaikh:** Formal analysis, Project administration. **Rafaqat Hussain Arain:** .

## Data Availability

Date Fruit Dataset for Inspection and Grading (Original data) (Mendeley Data).
